# Additive and multiplicative hazards modeling for recurrent event data analysis

**DOI:** 10.1186/1471-2288-11-101

**Published:** 2011-06-27

**Authors:** Hyun J Lim, Xu Zhang

**Affiliations:** 1 Department of Community Health & Epidemiology College of Medicine, University of Saskatchewan 107 Wiggins Road Saskatoon, SK S7N 5E5, Canada; 2 Department of Mathematics & Statistics Georgia State University 750 COE, 7th floor, 30 Pryor Street Atlanta, Georgia 30303, USA

## Abstract

**Background:**

Sequentially ordered multivariate failure time or recurrent event duration data are commonly observed in biomedical longitudinal studies. In general, standard hazard regression methods cannot be applied because of correlation between recurrent failure times within a subject and induced dependent censoring. Multiplicative and additive hazards models provide the two principal frameworks for studying the association between risk factors and recurrent event durations for the analysis of multivariate failure time data.

**Methods:**

Using emergency department visits data, we illustrated and compared the additive and multiplicative hazards models for analysis of recurrent event durations under (i) a varying baseline with a common coefficient effect and (ii) a varying baseline with an order-specific coefficient effect.

**Results:**

The analysis showed that both additive and multiplicative hazards models, with varying baseline and common coefficient effects, gave similar results with regard to covariates selected to remain in the model of our real dataset. The confidence intervals of the multiplicative hazards model were wider than the additive hazards model for each of the recurrent events. In addition, in both models, the confidence interval gets wider as the revisit order increased because the risk set decreased as the order of visit increased.

**Conclusions:**

Due to the frequency of multiple failure times or recurrent event duration data in clinical and epidemiologic studies, the multiplicative and additive hazards models are widely applicable and present different information. Hence, it seems desirable to use them, not as alternatives to each other, but together as complementary methods, to provide a more comprehensive understanding of data.

## 1. Background

Sequentially ordered multivariate failure time data or recurrent event time data are commonly observed in biomedical longitudinal studies. Examples include tumor recurrences, epileptic seizures, asthma attacks, migraines, infectious episodes, myocardial infarctions, injuries, and admissions to the hospital.

In general, standard hazard regression methods cannot be applied because of correlations between multivariate failure or recurrent event times within a subject. Adjustment is necessary for existing correlations, and more sophisticated analytic approaches are needed to obtain accurate estimates and efficient inferences. In the presence of the dependence between recurrent event times within a subject and subject-specific susceptibility across subjects, a variety of statistical methods have been proposed for the estimation of the covariate effect. In survival analysis, multiplicative and additive hazards models provide the two principal frameworks for studying the association between risk factors and recurrent event durations for the analysis of multivariate failure time data.

The majority of existing regression methods for analyzing multivariate failure or recurrent event time data assumes multiplicative covariate effects. Various authors have considered multivariate failure time models to be extensions of the Cox proportional hazards model [1]. The multivariate model with a Markov assumption, the conditional approach, the marginal approach, and the random effects approach are among them. Anderson and Gill proposed use of modeling under a Markov assumption [2]. Wei et al and Lee et al proposed use of the marginal approach [3,4]. Prentice et al proposed use of a semi-parametric model when multivariate failure times are conditionally independent, given the covariates [5]. Others used the random effect frailty model or the conditional frailty model for such recurrent event data analysis [6,7]. The popularity of these multiplicative models derives not only from their utility and wide applicability, but also from convention and the availability of statistical software. In general, consideration is not given to the possibility that the true underlying covariate effects may add to, rather than multiply, the baseline hazards. The semiparametric additive hazards model proposed by Lin and Ying [8] is the most closely connected analogue of the multiplicative Cox hazards model. Their additive hazards model assumes that covariates act in an additive manner on an unknown baseline hazard rate and that the effect of a covariate is time-invariant. Numerous authors advocated and utilized the additive hazards models for multivariate failure time data [9-14].

In this paper, we applied both multiplicative and additive models to the pediatric firearm victim's emergency department visit data. We considered the gap time model to be a multiplicative hazards model, as recommended for analysis of recurrent event time data by several authors [7,15]. We considered the Lin and Ying's model [10] to be an additive hazards model in our data analysis. The multiplicative and additive hazards models for analysis of recurrent event data with two scenarios were considered: (i) a varying baseline with a common coefficient effect and (ii) a varying baseline with an order-specific coefficient effect. The proposed models were applied to the emergency department (ED) visits of pediatric firearm victims, and difference between the models was examined.

Four additional sections comprise this paper. In Section 2, the models and methods for the analysis of recurrent event duration data are reviewed. In Section 3, a description of the ED visit study and the methods applied to this dataset are provided. Section 4 contains a discussion, in which the applicability and appropriateness of each model are discussed.

## 2. Methods

Within the framework of the multiplicative or additive hazards regression models, a variety of models have been proposed and utilized in real applications. Among the rich selection of different models, the gap time model as an extension of the multiplicative Cox proportional hazards model [5] and the Lin and Ying's additive model (L-Y model) [8,10] received the greatest attention due to easy interpretation of the covariate effects. These two models assume unspecified baseline hazards and constant covariate effects. In the models, we will assume that all censoring is non-informative and independent.

### 2.1. Basic Notations

Suppose that there are *n *subjects and that each subject can experience *K *failures or recurrent events. Suppose that censoring is non-informative, which means that knowledge of a censoring time for a subject provides no further information about the subject's likelihood of survival at a future time. Let *T*_*ik *_be the time when the *k*th failure occurs for the *i*th subject and *C*_*ik *_be the corresponding censoring time. *T*_*ik *_is measured from the subject's study enrollment and the censoring *C*_*ik *_occurs after the subject has been entered into a study to the right of the last known failure time; thus, it is right censoring. When *T*_*ik *_is subject to right censoring, the *k*th failure time *X*_*ik *_is a minimum of (*T*_*ik*_, *C*_*ik*_), i.e., *X*_*ik *_is equal to *T*_*ik *_if the event was observed and is equal to *C*_*ik *_if it is censored. Let *δ*_*ik *_= I(*T*_*ik *_≤ *C*_*ik*_), where I(.) is an indicator function and takes the value 1 when *T*_*ik *_≤ *C*_*ik *_and is 0 otherwise. Let *Z*_*ik *_be a covariate vector of *p*-dimensions for the *i*th subject at the *k*th failure. For each of the *K *failures, the hazard function for the *i*th subject with respect to the *k*th failure,*λ*_*ik *_(*t*), is assumed to take additive or multiplicative forms.

### 2.2. Multiplicative hazards model

The gap time model requires the same assumptions as the Cox proportional hazards model, but they allow the baseline hazard to vary from recurrence to recurrence. Gap time is defined the time between two successive failures experienced by the same subject [5]. For the gap time model, the hazard function is(1)

where *t *is the time since a patient's study enrollment and *t*_*k-1 *_is the time of the (*k-1*)th failure. Note that *λ*_0*k*_(*t*) are unspecified baseline hazard functions varying with *k *= 1, .., *K*. The corresponding partial likelihood function [16,17] is(2)

where *G*_*i,k *_= *X*_*ik *_*-X*_*i,k-1 *_is the inter-event or gap time interval and *Y*_*jk *_*(t) = I (G*_*ik *_≥ *t) *is a risk set indicator.  is a *p*-vector of regression coefficients of *Z*_*i*_.

In order to draw a semi-parametric inference on  for the model (1), the score functions  are obtained by differentiating the logarithm of  with respect to . The maximum partial likelihood estimator  is obtained by solving the corresponding score equation, . When failure times are independent, the variance-covariance matrix is estimated from the inverse of the information matrix, , called the "naïve" variance-covariance matrix; however, when failure times are dependent,  is not a good estimator of the variance-covariance matrix. When there are dependencies, the variance-covariance matrix , the so-called "sandwich" or "robust" variance-covariance estimator, is obtained from , where  is a data-based estimator, i.e., the cross-product of the empirical score residual matrix . Here, , and

where 

and

Therefore, it turns out that , which is called the "robust" variance-covariance estimate, and a detailed derivation is given by Wei et al [16] and Lin [17]. To account for the within-subject correlation, we used this robust "sandwich" method in the estimation of standard errors.

### 2.3. Additive hazards model

The additive hazards model is considered for multivariate survival data in which individuals may experience events of same or different types and in which there may also be correlation between individuals. A *p*-vector of the covariates *Z*_*ik *_in the additive hazards model acts additively on unknown baseline hazards, while it acts multiplicatively in the multiplicative model. The hazard function *λ*_*ik *_(*t*) for the *k*th gap time *t *since a patient's last failure is in a linear form,(3)

Where *λ*_0*k *_(*t*) is the unknown and unspecified baseline hazard function for the *k*th gap time and  is a *p *x 1 vector of the regression coefficients. When there is only one failure event (i.e., *K *= 1), the model (3) reduces to a univariate additive hazards model [8].

It turns out that a convenient representation of the data is given by the counting process, *N*_*ik*_(*t*). With the commonly employed counting process notation, an at-risk indicator is defined as *Y*_*ik*_*(t) *= I(*G*_*ik *_≤ *t*), and observed-event counting processes are defined as . The marginal filtration *F*_*ik *_*(t) *is defined by

By the Doob-Meyer decomposition,(4)

where *M*_*ik *_*(t) *is a local square-integrable martingale with respect to *F*_*ik *_*(t) *[2]. As a result of the underlying correlation, *M*_*ik *_*(t) *is not a martingale with respect to the joint filtration generated by all the failures, censoring, and covariates up to time *t *[18]. From (4),

If the denote the estimates of the true regression parameter , then under the working independent assumption, the cumulative baseline hazard function Λ_0*k*_(*t*) for the *k*th failure can be estimated by

Lin and Ying (1997) proposed to estimate  from the following estimating function

By substituting , the above function is equivalent to

where

The estimate of the model parameter  is obtained by solving the equation  and we obtain the consistent estimator ,

where for any vector *a*, *a*^⊗2 ^= *aa*^*T*^. The variance-covariance matrix of  may be estimated by A^-1 ^V A^-1^, where

and

Using empirical process theory, ***U ***() is shown to be a sum of independently, identically distributed random variables and thus follows a zero-mean Gaussian process by the functional central limit theorem; see Pollard [19], page 53, or van der Vaart & Weller [20], Section 2.11. Using Taylor's series expansion and some probability arguments,  converges in distribution to a zero-mean normal distribution [11].

## 3. Results

### 3.1. Study Description

The pediatric firearm victim's ED visit study was a retrospective cohort study. Data consisted of medical record reviews of follow-ups of firearm victims younger than 19-years-old who were presenting to the Pediatric Emergency Department/Trauma Center at the Children's Hospital of Wisconsin and all other hospitals in the Milwaukee metropolitan area between 1990 and 1997.

More detailed descriptions of the study design and profile have been published elsewhere [21,22]. Briefly, a total of 511 subjects were eligible for this study; this sample was taken from the pediatric firearm ED visit database. The events of interest are the times to ED revisit following the initial visit due to injury. Each subject experienced, at various times, a varying number of visits to the ED, which represent the whole observable history of his/her recurrences. Each subject has a number of ED visits and contributes several observations, which are dependent when there is inter-subject variation. Of these 511 subjects, 263 (51.5%) had at least one ED revisit during the follow-up period (median follow-up time = 3.2 years). Table 1 summarizes the number of events experienced by the 511 subjects during the follow-up period. A total of 571 occurrences of ED revisits were observed, with some persons experiencing a rather large number (up to six revisits). In our study, any ED revisit due to injuries was defined as a recurrent event, and up to four revisits per subject were used in the subsequent analysis, as too few subjects experienced more than four revisits. The main purpose of this study, however, was to illustrate the application of the multiplicative and additive hazards models to recurrent event duration data and provide comparisons between the models, rather than give universally valid estimates for ED revisits in a pediatric population.

**Table 1:** Number of revisits to the emergency department.

Number of events	0	1	2	3	4	5	6	Total
**Number of subjects (%)**	248 (48.5)	130 (25.4)	52 (10.2)	30 (5.9)	25 (4.9)	9 (1.8)	17 (3.3)	511 (100)

### 3.2. Analysis of the dataset

We applied the multiplicative and additive hazards described in Section 2 to the ED visit gap time data, allowing a varying baseline with common coefficient effects and with order-specific coefficient effects, respectively. The adequacy of the models was assessed by residuals and Arjas plots. The ED revisit was defined as the recurrent event. When a subject is already in the ED, the subject is not at the risk for an ED revisit. Four baseline characteristics -- age, gender (male ≡ 1; female ≡ 0), race/ethnicity (black ≡ 1; others ≡ 0), and parents (1 if subject had parents as guardian; 0 otherwise) -- were included in the models. We fitted the multiplicative gap time model and the L-Y additive hazards model. When we assume that the regression parameters were similar for all ED revisits or are interested in global covariate effects, we can adopt a model with a common covariate effect, i.e.,  for all *k *= 1, .., *K*. Table 2 shows the coefficient estimates and standard errors of the common covariate effects. When we assume that the regression parameters are different for each ED revisit order, we can use a model with an order-specific covariate effect. Table 3 shows the coefficients estimates and standard errors of the order-specific covariate effects.

**Table 2:** Additive and multiplicative hazards models for recurrent event time, with a varying baseline and common coefficient effect.

*Model*	*Covariate*	*Estimate*	*S.E*	*Chi-square*	*p-value*
**Additive Hazards Model**	Gender ^#^	- 0.152	0.034	19.7	<0.0001
	Race/ethnicity ^&^	0.058	0.027	4.71	0.030
	Age	0.009	0.003	6.34	0.012
	Parent ^@^	0.033	0.024	1.91	0.167
**------------------**	----------------	---------	--------	--------	--------
**Multiplicative Hazards Model**	Gender	-0.654	0.139	35.4	<0.0001
	Race/ethnicity	0.335	0.148	6.31	0.012
	Age	0.043	0.016	8.22	0.004
	Parent	0.152	0.117	2.37	0.124

Estimation of regression coefficients with "robust" standard errors (S.E.), chi-squares, and p-values.

^# ^gender ≡ 1 if subject is male and 0 if otherwise.

^&^race/ethnicity ≡ 1 if subject is Black and 0 if otherwise.

^@ ^parent ≡ 1 if subject had parents or a single parent as guardian(s), and 0 if otherwise.

**Table 3:** Recurrent event time models with varying baseline and order-specific coefficient effects, from the additive and multiplicative hazards models.

Model	Order	Covariate	Estimate	S.E	Chi-square	p-value
**Additive Hazards Model**	1	Gender ^#^	-0.103	0.06	2.96	0.085
		Race/ethnicity ^&^	0.068	0.036	3.66	0.056
		Age	0.004	0.005	0.60	0.439
		Parent ^@^	0.063	0.034	6.70	0.065
						
	2	Gender	-0.202	0.067	9.13	0.003
		Race/ethnicity	0.038	0.051	0.55	0.457
		Age	0.015	0.005	7.94	0.005
		Parent	0.028	0.049	0.33	0.567
						
	3	Gender	-0.181	0.053	11.7	0.001
		Race/ethnicity	0.003	0.078	0.002	0.967
		Age	0.011	0.008	1.92	0.165
		Parent	-0.025	0.046	0.30	0.583
						
	4	Gender	-0.158	0.013	2.65	0.103
		Race/ethnicity	0.068	0.121	0.32	0.571
		Age	0.021	0.013	2.65	0.104
		Parent	-0.075	0.088	0.73	0.394
---------------------	-------	---------------	------------	------------	-----------	----------
						
**Multiplicative Hazards Model**	1	Gender	-0.464	0.179	7.36	0.007
		Race/ethnicity	0.391	0.175	5.05	0.025
		Age	0.021	0.021	1.15	0.285
		Parent	0.347	0.144	5.99	0.014
						
	2	Gender	-0.864	0.235	15.7	< 0.0001
		Race/ethnicity	0.185	0.262	0.45	0.505
		Age	0.078	0.029	6.33	0.012
		Parent	0.112	0.215	0.31	0.577
						
	3	Gender	-0.747	0.25	8.21	0.004
		Race/ethnicity	0.085	0.445	0.04	0.842
		Age	0.051	0.047	1.03	0.309
		Parent	-0.138	0.248	0.29	0.589
						
	4	Gender	-0.732	0.28	5.44	0.02
		Race/ethnicity	0.357	0.469	0.48	0.49
		Age	0.103	0.073	1.71	0.191
		Parent	-0.31	0.323	0.89	0.345

Estimation of regression coefficients, "robust" standard errors (S.E.), chi-square, and p-values.

* guardian ≡ 1 if subject had parents or a single parent as guardian(s), and 0 if otherwise.

^&^race/ethnicity ≡ 1 if subject is Black and 0 if otherwise.

^# ^gender ≡ 1 if subject is male and 0 if otherwise.

The estimates from the additive and multiplicative hazards models had the same signs, indicating the same directions of the covariate effects. The standard errors from the additive hazard model were smaller than those of the multiplicative model. However, while the p-values for the two models differ, the inferences were consistent. Almost all of the models from both the common and order-specific covariate effects showed that gender was the significant risk factor for ED revisits.

Both additive and multiplicative hazards models with varying baseline and common coefficient effects gave similar results with regard to covariates selected to remain in the model (Tables 2). Three covariates showed significant impact on ED revisits in both hazards models: gender, race/ethnicity, and age. The result obtained under the additive model with a varying baseline and common coefficient effect in Table 2 suggested that females tended to have more-delayed ED revisits, compared to males, and being older and black were associated with significantly shorter gap times. On the other hand, the gap time did not seem to be related to having a parent as a guardian; the p-values were 0.167 and 0.124 for the additive and multiplicative hazards model, respectively. Estimates of the ED revisit order-specific covariate effects for the models with varying baselines are shown in Table 3. For all orders, *k *= 1, .., 4, gender was again the only significant risk factor for an ED revisit in the multiplicative hazards model, but this was not true for the additive hazards model. In both models, age was significant for the 2^nd ^revisit but not for any other revisit. Ethnicity and having a parent as a guardian were not significant in the additive model for all *k*.

**Figure 1:**
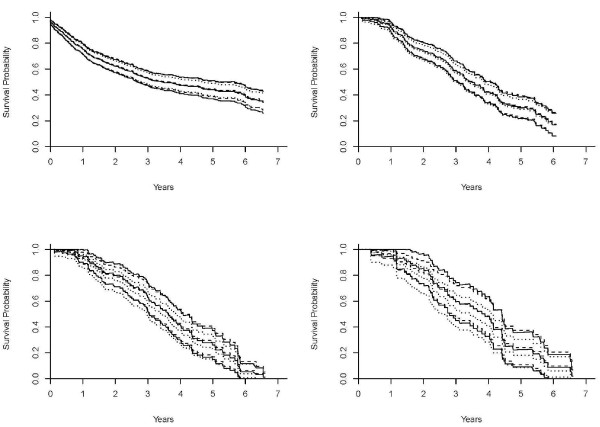
**Estimates of the survival curves of the models with a varying baseline and common coefficient effect**. Estimates of the survival curves for a 15-year-old, black male who has parents as guardians and no previous injury history, under the multiplicative hazards model (dashed curve), Lin & Ying's additive hazards model (solid curve), and the Kaplan-Meier (dotted curve) with a varying baseline and common coefficient effect for the revisit *k *= 1 (1a: top left), *k *= 2 (1b: top right), *k *= 3 (1c: bottom left), and *k *= 4 (1d: bottom right).

**Figure 2:**
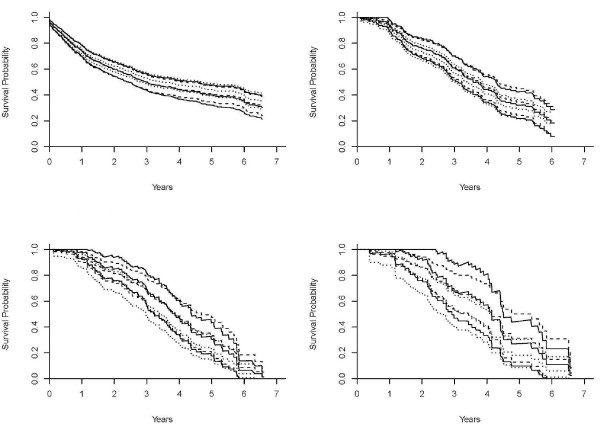
**Estimates of the survival curves of the models with a varying baseline and order-specific coefficient effects**. Estimates of the survival curves for a 15-year-old, black male who has parents as guardians and no previous injury history, under the multiplicative hazards model (dashed curve), Lin & Ying's additive hazards model (solid curve), and the Kaplan-Meier (dotted curve) with a varying baseline and revisit order-specific coefficient effects for the revisit *k *= 1 (2a: top left), *k *= 2 (2b: top right), *k *= 3 (2c: bottom left), and *k *= 4 (2d: bottom right).

**Figure 3:**
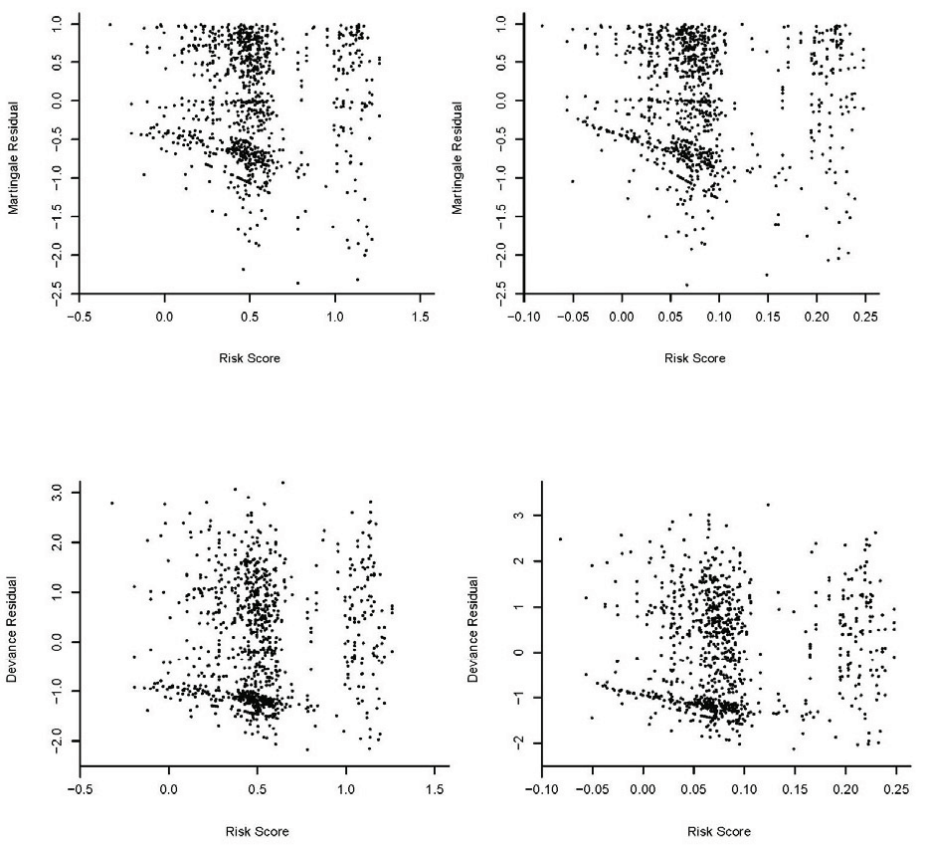
**Residuals Plots**. Martingale residuals for the multiplicative model (3a: top left) and for the additive model (3b: top right). Deviance residuals for the multiplicative model (3c: bottom left) and for the additive model (3d: bottom right).

To illustrate the prediction of the survival probability for a given subject, Figures 1a-d and 2a-d (a dashed curve for the multiplicative hazards model; a solid curve for the L-Y additive hazards model) showed the estimated survival curves for a 15-year-old patient, a black male with a parent as guardian, under the multiplicative and additive hazards models with a varying baseline and common coefficient effect. The selected covariate values were roughly the sample median. Figures 1a-d show the estimated survival functions, based on the additive and multiplicative hazards models with a varying baseline and common coefficient effect were very similar. Comparing to the Kaplan-Meier estimate, the estimates of both additive and multiplicative hazards models were larger than the K-M estimate for all *k*. However, the differences of the estimate increased as the order increased. Figures 2a-d show the estimated survival curves based on the additive and multiplicative hazards models, with a varying baseline and order-specific coefficient effect, for each order *k *= 1, .., 4. For *k *= 1, 2, 3, the survival curves of these two models were almost identical. For the higher revisit orders (Figures 2c-d), the multiplicative hazards model had slightly higher survival curves than the survival curves estimated under the additive hazards model. The confidence intervals of the additive and multiplicative hazards models were similar except the order 4 and get wider as the revisit order increases because the risk set decreased as the revisit order increased. All these facts concerning the prediction of the survival probability were consistently observed when the values of the covariates were changed. The martingale and deviance residuals for these two models with common coefficient effect showed that the models fit well (Figure 3a-d). The residuals plots and the Arjas plots of the covariates for the models with a varying baseline and order-specific covariate effect showed that the models also fit well. (figures not shown). For all estimations in this study, we used SAS PHREG procedure to fit the multiplicative model and a SAS macro for additive model. All codes for the programs can be found in Appendix.

## 4. Discussion

Among recurrent event data, correlation between event durations within a subject exists. For an example, one can suppose that more frequently a subject experiences episodes of injury, the sooner the next injury is likely to occur. In this study, the additive and multiplicative hazards regression models for the recurrent event duration analysis were examined and illustrated with a real dataset. Differences in estimates from the models under (i) a varying baseline with a common covariate effect and (ii) a varying baseline with an order-specific covariate effect were compared using the pediatric firearm victim's ED visit data. The additive and multiplicative models revealed similar results with regard to covariates selected to remain in the model: gender, race/ethnicity, and age. The estimated survival functions, based on the additive and multiplicative hazards models from our data, were similar. Our example showed that the goodness-of-fit of both multiplicative and additive hazards models was satisfactory.

The standard errors increase as the order increases because the size of the risk set for the models decreases after each revisit. If the risk set decreases rapidly, then it yields estimates that are less reliable with a small risk set size. However, in our study, the standard errors increased moderately. The coefficients of the models cannot be compared directly because the coefficients of the former act in a multiplicative way on an unknown baseline hazard, whereas the coefficients of the latter act in an additive way on unknown baseline hazard or represent coefficient function for added risks. A naïve way of comparing the models would be comparing p-values, which would indicate the power of rejecting the null hypothesis for selected covariate in the models. In order to detect any difference between models in terms of prediction, a comparison between two model-based survival curves with the nonparametric estimate of the survival function could be performed. In our study, the survival curves of these two models were larger than the Kaplan-Meier estimate for all order, but the differences were negligible.

The additive model is plausible for many applications and is often attractive in epidemiologic applications, for example, a study of diabetic patients [23]. In such a study, λ_0 _is taken to be the baseline mortality of the standard population and *β *measures excess risk for the patients under study. The excess mortality is more appealing than the relative mortality to provide an inference on how the study population' mortality differs from that of the standard population.

The additive and multiplicative hazards models can capture the risk process for patients with average comorbidity profiles equally well. In cases where both the additive and multiplicative models fit the data fairly well, an additive specification may be preferred, due to the interpretation of the regression parameters. One of the major advantages of using the additive hazards model over the multiplicative hazards model is that the resulting regression parameter estimator has a closed form [13]. Regression coefficients from the additive model are more interpretable in public health research since they represent differences in event rates, as opposed to ratio [24]. A practical drawback of using the additive models is that the current standard procedure for fitting additive models is still limited, whereas statistical software for the multiplicative model is available and easy to use.

In the presence of the dependence between recurrent events in multivariate failure time data, frailty model have been proposed for the estimation of the covariate effect by incorporation of additional unobserved random frailty effects into standard survival models [6]. The covariate estimates in the frailty model are estimated conditionally on the unobservable frailty, and because of this, their interpretation is often ambiguous [7]. When the primary interest of investigation is a measurement of the dependence of correlated repeated events within a subject, the frailty model approach is adequate [25]. However, our study does not focus on the dependence measurement of recurrent events.

Recurrent event duration data are the archetypical example of series data, which differ from parallel multivariate failure time data. Because the study period is typically less than the first failure time, the marginal distribution of the second gap time is not identifiable unless within-subject failure times are independent. Even if the total times are censored independently, the subsequent failure times will be subject to induced dependent censoring [17,18]. To analyze such recurrent event duration data, the non-informative censoring assumption is required for the validity of the statistical analysis. However, when the recurrence is influencing a censoring mechanism such as dropout or death, censoring is informative about the event process; therefore, the non-informative censoring assumption is violated, and subjects in the risk set do not form a representative sample from the target population. An important assumption of the models examined in this paper is that the recurrent event process is independent of the censoring process. Suitable modification of the methodologies needs to be further studied to adjust for such informative censoring mechanisms related to terminal events in the recurrent event analysis [26]. In addition, there is relatively little information in the literature on the goodness-of-fit for multiple failure time models.

## 5. Conclusion

In this study, we illustrated and compared the additive and multiplicative hazards models for analysis of recurrent event durations. In summary, the choice between the additive and multiplicative models will typically be an empirical matter. Due to the frequency of recurrent event duration data in clinical and epidemiologic studies, the proposed additive and multiplicative methods are widely applicable. The two modeling approaches have sound biological bases, providing complementary information about the association between risk factors and death. An overall conclusion is that the additive and multiplicative hazards models present different aspects of the association between risk factors and the event durations. Hence, two hazards models give different information and it seems desirable to use them -- not as alternatives to each other, but as complementary methods -- together to gain a more comprehensive understanding of the data. Practitioners may benefit from the use of these statistical models, which help in predicting the effect of one or more variables and in verifying their influence on study outcomes.

## Competing interests

The authors declare that they have no competing interests.

## Authors' contributions

HJL conducted the literature review, developed the mathematical framework, derived the results, and prepared the manuscript. XZ wrote computer programs and produced graphs.

All authors have read and approved the final manuscript.

## Appendix

Suppose a subject has four recurrent events, say at *t*_*1*_, *t*_*2*_, *t*_*3*_, and *t*_*4*_. For the model analysis the subject is represented as a set of rows with time intervals of (0, *t*_*1*_], (0, *t*_*2 *_- *t*_*1*_], (0, *t*_*3 *_- *t*_*2*_], and (0, *t*_*4 *_- *t*_*3*_] for the gap time. Letting *gap *be equal to *t*_*k *_- *t*_*k-1 *_for *k *= 1, .. 4, then the following programs specify the models.

### A1. *Data Management *for recurrent events

data counting;

set firearm;

gap = tstop-tstart;

bguardn1 = 0;

if visit = 1 then bguardn1 = bguardn;

bguardn2 = 0;

if visit = 2 then bguardn2 = bguardn;

bguardn3 = 0;

if visit = 3 then bguardn3 = bguardn;

bguardn4 = 0;

if visit = 4 then bguardn4 = bguardn;

bracen1 = 0;

if visit = 1 then bracen1 = bracen;

bracen2 = 0;

if visit = 2 then bracen2 = bracen;

bracen3 = 0;

if visit = 3 then bracen3 = bracen;

bracen4 = 0;

if visit = 4 then bracen4 = bracen;

bage1 = 0;

if visit = 1 then bage1 = bage;

bage2 = 0;

if visit = 2 then bage2 = bage;

bage3 = 0;

if visit = 3 then bage3 = bage;

bage4 = 0;

if visit = 4 then bage4 = bage;

sexn1 = 0;

if visit = 1 then sexn1 = sexn;

sexn2 = 0;

if visit = 2 then sexn2 = sexn;

sexn3 = 0;

if visit = 3 then sexn3 = sexn;

sexn4 = 0;

if visit = 4 then sexn4 = sexn;

run;

### A2. *SAS PHREG *procedure for multiplicative model

title 'Gap Time Multiplicative Model with different betas and different baselines;

proc phreg data = counting;

model gap*status(0) = guard1 guard2 guard3 guard4 race1 race2 race3 race4

sex1 sex2 sex3 sex4;

strata visit;

id IDnumber;

run;

title 'Gap Time Multiplicative Model: common beta and different baselines';

proc phreg data = counting covs(aggregate);

model gap*status(0) = guard race sex;

strata visit;

id IDnumber;

run;

### A3. *SAS Macro *for additive model

%*est*(counting, gap, fail, visit, 4, id, bguardn bracen sexn bage);

proc transpose data = best out = best;

proc transpose data = se out = se;

proc transpose data = wlwse out = wlwse; run;

data item;

Variable = 'bguardn'; output;

Variable = 'bracen'; output;

Variable = 'sexn'; output;

Variable = 'bage'; output;

run;

data all;

merge item best (rename = (col1 = Estimate))

se (rename = (col1 = Naive_SE))

wlwse (rename = (col1 = Sandwich_SE));

Chisq = (Estimate/Sandwich_SE)**2;

Prob = 1-probchi(Chisq,1);

drop _name_;

run;

title 'Additive model - gap time';

title2 'common beta, different baselines';

proc print data = all; run;

%*est*(jing2, gap, fail, visit, 4, id,

bguardn1 bracen1 sexn1 bage1 bguardn2 bracen2 sexn2 bage2 bguardn3 bracen3 sexn3 bage3 bguardn4 bracen4 sexn4 bage4);

proc transpose data = best out = best;

proc transpose data = se out = se;

proc transpose data = wlwse out = wlwse; run;

data item;

Variable = 'bguardn1'; output;

Variable = 'bracen1'; output;

Variable = 'sexn1'; output;

Variable = 'bage1'; output;

Variable = 'bguardn2'; output;

Variable = 'bracen2'; output;

Variable = 'sexn2'; output;

Variable = 'bage2'; output;

Variable = 'bguardn3'; output;

Variable = 'bracen3'; output;

Variable = 'sexn3'; output;

Variable = 'bage3'; output;

Variable = 'bguardn4'; output;

Variable = 'bracen4'; output;

Variable = 'sexn4'; output;

Variable = 'bage4'; output;

run;

data all;

merge item best (rename = (col1 = Estimate))

se (rename = (col1 = Naive_SE))

wlwse (rename = (col1 = Sandwich_SE));

Chisq = (Estimate/Sandwich_SE)**2;

Prob = 1-probchi(Chisq,1);

drop _name_;

run;

title 'Additive model - gap time';

title2 'different betas, different baselines';

proc print data = all; run;

## Pre-publication history

The pre-publication history for this paper can be accessed here:

http://www.biomedcentral.com/1471-2288/11/101/prepub

## References

[B1] Cox DR Regression models and life-tables (with discussion) Journal of the Royal Statistical Society B 1972 34 187 220

[B2] Anderson PK Gill RD Cox's regression model for counting processes: A large sample study The Annals of Statistics 1982 10 1100 1120 10.1214/aos/1176345976

[B3] Wei LJ Lin DY Weissfeld L Regression analysis of multivariate incomplete failure time data by modeling marginal distributions Journal of American Statistical Association 1989 84 1065 1073 10.2307/2290084

[B4] Lee EW Wei LJ Amato DA JP Klein, PK Goel Cox-type regression analysis for large number of small groups of correlated failure time observations Survival Analysis: State of the Art 1992 Dordrecht: Kluwer Academic Publisher

[B5] Prentice RL Williams BJ Peterson AV On the regression analysis of multivariate failure time data Biometrika 1981 68 373 379 10.1093/biomet/68.2.373

[B6] Oakes D JP Klein, PK Goel Frailty models for multiple event times Survival Analysis: State of the Art 1992 Dordrecht: Kluwer Academic Publisher

[B7] Box-Steffensmeier JM Suzanna DB Repeated events survival models: The conditional frailty model Statistics in Medicine 2006 25 3518 33 10.1002/sim.2434 16345026

[B8] Lin DY Ying Z Semiparametric analysis of the additive risk model Biometrika 1994 81 61 71 10.1093/biomet/81.1.61

[B9] McKeague IW Asymptotic theory for weighted least squares estimators in Aalen's additive risk model Contemporary Mathematics 1988 80 139 152

[B10] Lin DY Ying Z Lin DY, Fleming TR Additive regression models for survival data Proceedings of the First Seattle Symposium in Biostatistics: Survival Analysis 1997 Springer: New York 185 198

[B11] Yin G Cai J Additive hazards model with multivariate failure time data Biometrika 2004 91 801 818 10.1093/biomet/91.4.801

[B12] Schaubel DE Cai J Analysis of clustered recurrent event data with application to hospitalization rates among renal failure patients Biostatistics 2005 6 404 419 10.1093/biostatistics/kxi018 15831581

[B13] Schaubel DE Zeng D Cai J A semiparametric additive rate model for recurrent event data Lifetime Data Anal 2006 12 389 406 10.1007/s10985-006-9017-x 17031499

[B14] Sun L Park D Sun G The additive hazards model for recurrent gap times Statistica Sinica 2006 16 919 932

[B15] Kelly PJ Lim LY Survival analysis for recurrent event data: an application to childhood infectious diseases Statistics in Medicine 2000 19 13 33 10.1002/(SICI)1097-0258(20000115)19:1<13::AID-SIM279>3.0.CO;2-5 10623910

[B16] Wei LJ Lin DY Weissfeld L Regression analysis of multivariate incomplete failure time data by modeling marginal Journal of the American Statistical Association 1989 84 1065 1073 10.2307/2290084

[B17] Lin DY Cox regression analysis of multivariate failure time data: the marginal approach Statistics in Medicine 1994 13 2233 2247 10.1002/sim.4780132105 7846422

[B18] Andersen PK Borgan Ø Gill RD Keiding N Statistical Models Based on Counting Processes 1993 Springer: New York

[B19] Pollard D Empirical Processes: Theory and Applications Regional Conference Series in Probability and Statistics V.2 1990 Institute of Mathematical Statistics, Howard, California

[B20] Van Der Vaart AW Weller JA Weak convergence and empirical processes 1996 New York: Springer

[B21] Marcelle DR Melzer-Lange MD Project UJIMA: Working together to make things right Wisconsin Medical Journal 2001 100 222 5 11419365

[B22] Lim HJ Liu J Melzer-Lange M Comparison of methods for analyzing recurrent event data: application to emergency department visits of pediatric firearm victims Accident Analysis & Prevention 2007 39 290 299 10.1016/j.aap.2006.07.009 17045949

[B23] Rosato R Ciccone G Pagano FG Gregori D Evaluating cadiovascular mortality in type 2 diabetes patient: an analysis based on competing risks Makov chains and additive regression models J of Eval in Clinical Practice 2007 13 422 428 10.1111/j.1365-2753.2006.00732.x 17518809

[B24] Lim HJ Zhang X Semi-parametric additive risk models: application to injury duration study Accident Analysis & Prevention 2009 41 211 216 10.1016/j.aap.2008.07.015 19245877

[B25] Clayton D Cuzick J Multivariate generalizations of the proportional hazards model Journal of the Royal Statistical Society A 1985 148 82 117 10.2307/2981943

[B26] Wang MC Chiang CT Nonparametric method for recurrent event data with informative and non- censorings Statistics in Medicine 2002 21 445 456 10.1002/sim.1029 11813230

